# (*E*)-1-(4-Bromo­phen­yl)ethan-1-one semicarbazone

**DOI:** 10.1107/S1600536809022284

**Published:** 2009-06-17

**Authors:** Hoong-Kun Fun, Jia Hao Goh, Mahesh Padaki, Shridhar Malladi, Arun M. Isloor

**Affiliations:** a X-ray Crystallography Unit, School of Physics, Universiti Sains Malaysia, 11800 USM, Penang, Malaysia; b Department of Chemistry, National Institute of Technology-Karnataka, Surathkal, Mangalore 575 025, India

## Abstract

In the title compound, C_9_H_10_BrN_3_O, the hydrazone portion and aliphatic chain are essentially coplanar [maximum deviation 0.057 (15) Å] and the mean plane makes a dihedral angle of 70.9 (6)° with the benzene ring. The main feature of the crystal structure is the inter­molecular N—H⋯O hydrogen bond, which links mol­ecules into zigzag chains along the *a* axis. These chains are further stacked along the *b* axis. The crystal structure features non-classical inter­molecular C—H⋯O inter­actions. The crystal studied was a nonmerohedral twin, with a twin ratio of 0.505 (1):0.495 (1).

## Related literature

For general background and applications of semicarbazone derivatives, see: Chandra & Gupta (2005 ▶); Jain *et al.* (2002 ▶); Pilgram (1978 ▶); Warren *et al.* (1977 ▶); Yogeeswari *et al.* (2004 ▶). For the preparation, see: Furniss *et al.* (1978 ▶). For bond-length data, see: Allen *et al.* (1987 ▶). For a related structure, see: Fun *et al.* (2009 ▶). For the stability of the temperature controller used for the data collection, see: Cosier & Glazer (1986 ▶).
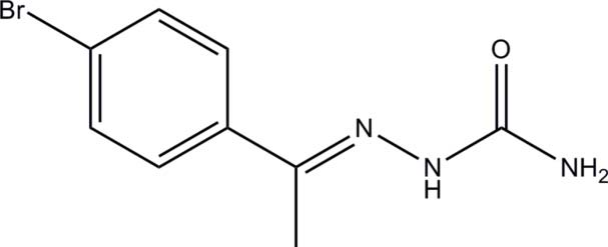


## Experimental

### 

#### Crystal data

C_9_H_10_BrN_3_O
*M* *_r_* = 256.11
Monoclinic, 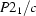

*a* = 17.6700 (8) Å
*b* = 7.3426 (4) Å
*c* = 7.9082 (4) Å
β = 102.953 (3)°
*V* = 999.93 (9) Å^3^
*Z* = 4
Mo *K*α radiation
μ = 4.08 mm^−1^
*T* = 100 K
0.22 × 0.12 × 0.08 mm

#### Data collection

Bruker SMART APEXII CCD area-detector diffractometer
Absorption correction: multi-scan (**SADABS**; Bruker, 2005 ▶) *T*_min_ = 0.466, *T*_max_ = 0.733
12017 measured reflections
2945 independent reflections
2105 reflections with *I* > 2σ(*I*)
*R*_int_ = 0.053

#### Refinement

*R*[*F* ^2^ > 2σ(*F* ^2^)] = 0.080
*wR*(*F* ^2^) = 0.249
*S* = 1.16
2945 reflections
129 parameters
1 restraint
H-atom parameters constrained
Δρ_max_ = 3.03 e Å^−3^
Δρ_min_ = −1.11 e Å^−3^

### 

Data collection: *APEX2* (Bruker, 2005 ▶); cell refinement: *SAINT* (Bruker, 2005 ▶); data reduction: *SAINT*; program(s) used to solve structure: *SHELXTL* (Sheldrick, 2008 ▶); program(s) used to refine structure: *SHELXTL*; molecular graphics: *SHELXTL*; software used to prepare material for publication: *SHELXTL* and *PLATON* (Spek, 2009 ▶).

## Supplementary Material

Crystal structure: contains datablocks global, I. DOI: 10.1107/S1600536809022284/ng2596sup1.cif

Structure factors: contains datablocks I. DOI: 10.1107/S1600536809022284/ng2596Isup2.hkl

Additional supplementary materials:  crystallographic information; 3D view; checkCIF report

## Figures and Tables

**Table 1:** Hydrogen-bond geometry (Å, °)

*D*—H⋯*A*	*D*—H	H⋯*A*	*D*⋯*A*	*D*—H⋯*A*
N2—H2*B*⋯O1^i^	0.86	2.21	3.052 (12)	168
N3—H3*A*⋯O1^ii^	0.86	2.03	2.885 (13)	171
C9—H9*A*⋯O1^iii^	0.96	2.51	3.34 (2)	144

Symmetry codes: (i) 
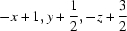
; (ii) 
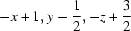
; (iii) 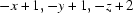.

**Fig. 1.**
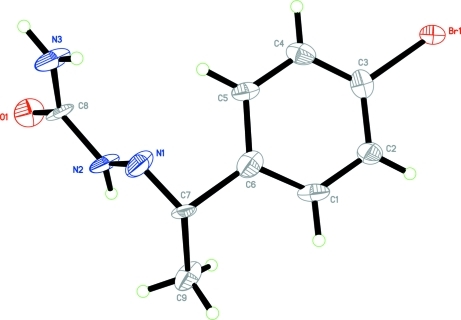
The molecular structure of the title compound, showing 50% probability displacement ellipsoids and the atom-numbering scheme.

**Fig. 2.**
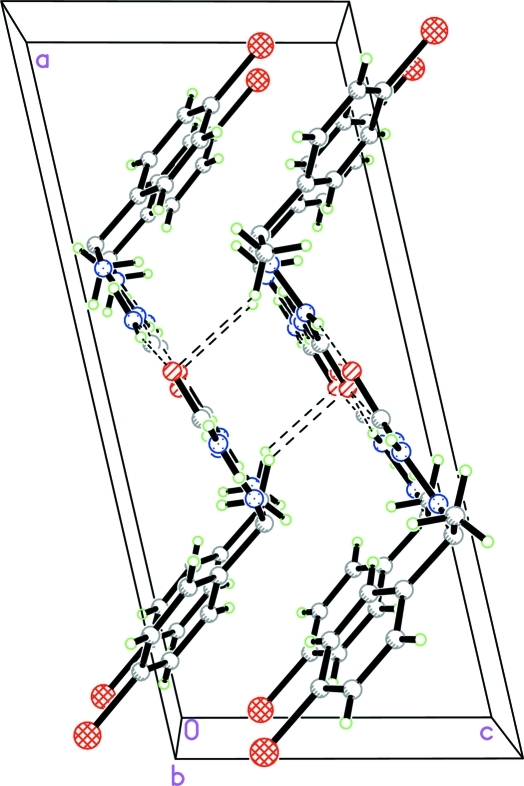
The crystal packing of the title compound, viewed along the b axis. Intermolecular interactions are shown as dashed lines.

## supplementary crystallographic information

### Comment

In organic chemistry, a semicarbazone is a derivative of an aldehyde or ketone formed by a condensation between a ketone or aldehyde and semicarbazide. Semicarbazones find immerse applications in the field of synthetic chemistry, such as in medicinal chemistry (Warren *et al.*, 1977), organometalics (Chandra & Gupta, 2005), polymers (Jain *et al.*, 2002) and herbicides (Pilgram, 1978). 4-Sulphamoylphenyl semicarbazones were synthesized and were found to posses anticonvulsant activity (Yogeeswari *et al.*, 2004). We hereby report the crystal structure of the semicarbazone of commercial importance keeping in view of their synthetic importance.

The bond lengths (Allen *et al.*, 1987) and angles in the molecule (Fig. 1) are within normal ranges and are comparable to a closely related structure (Fun *et al.*, 2009). Atoms C7, C8, N1, N2, N3 and O1 lie on the same plane with a maximum deviation of 0.057 (15) Å for atom N1. This plane makes dihedral angle of 70.9 (6)° with the C1-C6 benzene ring.

In the crystal packing (Fig. 2), N3—H3A···O1 hydrogen bonds link the molecules into one-dimensional zig-zag extended chains along the *a* axis. These chains are further stacked along the *b* axis and thus forming two-dimensional extended networks parallel to the *ab* plane. The crystal structure is further stabilized by intermolecular C9—H9A···O1 interactions.

### Experimental

0.57 g (5.11 mmol) of semicarbazide hydrochloride and 0.54 g (6.60 mmol) of crystallized sodium acetate was dissolved in 10 ml of water (Furniss *et al.*, 1978). The reaction mixture was stirred at room temperature for 10 minutes. 1 g (5.02 mmol) 4-Bromoacetophenone was added to this and shaken well. A little alcohol was added to dissolve the turbidity. It was shaken for 10 more minutes and allowed to stand. The semicarbazone crystallizes on standing for 6 h. The separated crystals were filtered, washed with cold water and recrystallized from alcohol. Yield was found to be 1.158 g, 90.05 %. *M.p.* 479-481 K.

### Refinement

The H-atoms bound to N2 and N3 was located from the difference Fourier map and refined freely. The rest of the hydrogen atoms were placed in calculated positions, with C—H = 0.93 Å, *U*_iso_ = 1.2*U*_eq_(C) for aromatic, and C—H = 0.96 Å, *U*_iso_ = 1.5*U*_eq_(C) for CH_3_ atom. A rotating group model was used for the methyl group. The N1—N2 bond was restrained with a N—N bond distance of 1.37 (1) Å. The crystal studied was a twin with the refined BASF parameter of 0.495 (1).

The final difference Fourier map had a peak/hole in the vicnity of Br1.

### Crystal data

**Table:**

C_9_H_10_BrN_3_O	*F*(000) = 512
*M**_r_* = 256.11	*D*_x_ = 1.701 Mg m^−^^3^
Monoclinic, *P*2_1_/*c*	Mo *K*α radiation, λ = 0.71073 Å
Hall symbol: -P 2ybc	Cell parameters from 3514 reflections
*a* = 17.6700 (8) Å	θ = 3.0–28.2°
*b* = 7.3426 (4) Å	µ = 4.08 mm^−^^1^
*c* = 7.9082 (4) Å	*T* = 100 K
β = 102.953 (3)°	Block, colourless
*V* = 999.93 (9) Å^3^	0.22 × 0.12 × 0.08 mm
*Z* = 4	

### Data collection

**Table:**

Bruker SMART APEXII CCD area-detector diffractometer	2945 independent reflections
Radiation source: fine-focus sealed tube	2105 reflections with *I* > 2σ(*I*)
graphite	*R*_int_ = 0.053
φ and ω scans	θ_max_ = 30.2°, θ_min_ = 1.2°
Absorption correction: multi-scan (*SADABS*; Bruker, 2005)	*h* = −24→24
*T*_min_ = 0.466, *T*_max_ = 0.733	*k* = −10→10
12017 measured reflections	*l* = −11→11

### Refinement

**Table:**

Refinement on *F*^2^	Primary atom site location: structure-invariant direct methods
Least-squares matrix: full	Secondary atom site location: difference Fourier map
*R*[*F*^2^ > 2σ(*F*^2^)] = 0.080	Hydrogen site location: inferred from neighbouring sites
*wR*(*F*^2^) = 0.249	H-atom parameters constrained
*S* = 1.16	*w* = 1/[σ^2^(*F*_o_^2^) + (0.1217*P*)^2^ + 9.4338*P*] where *P* = (*F*_o_^2^ + 2*F*_c_^2^)/3
2945 reflections	(Δ/σ)_max_ < 0.000
129 parameters	Δρ_max_ = 3.03 e Å^−^^3^
1 restraint	Δρ_min_ = −1.11 e Å^−^^3^

### Special details

**Table:**

Experimental. The crystal was placed in the cold stream of an Oxford Cyrosystems Cobra open-flow nitrogen cryostat (Cosier & Glazer, 1986) operating at 100.0 (1)K.
Geometry. All esds (except the esd in the dihedral angle between two l.s. planes) are estimated using the full covariance matrix. The cell esds are taken into account individually in the estimation of esds in distances, angles and torsion angles; correlations between esds in cell parameters are only used when they are defined by crystal symmetry. An approximate (isotropic) treatment of cell esds is used for estimating esds involving l.s. planes.
Refinement. Refinement of F^2^ against ALL reflections. The weighted R-factor wR and goodness of fit S are based on F^2^, conventional R-factors R are based on F, with F set to zero for negative F^2^. The threshold expression of F^2^ > 2sigma(F^2^) is used only for calculating R-factors(gt) etc. and is not relevant to the choice of reflections for refinement. R-factors based on F^2^ are statistically about twice as large as those based on F, and R- factors based on ALL data will be even larger.

### Fractional atomic coordinates and isotropic or equivalent isotropic displacement parameters (Å^2^)

**Table:**

	*x*	*y*	*z*	*U*_iso_*/*U*_eq_	
Br1	0.03467 (5)	0.43707 (10)	0.2675 (2)	0.0212 (2)	
N3	0.4198 (6)	0.1238 (13)	0.8352 (16)	0.034 (2)	
H3A	0.4430	0.0237	0.8214	0.041*	
H3B	0.3772	0.1214	0.8704	0.041*	
N1	0.3450 (5)	0.4205 (11)	0.899 (2)	0.039 (3)	
N2	0.4105 (5)	0.4401 (11)	0.8290 (14)	0.027 (2)	
H2B	0.4257	0.5459	0.8038	0.032*	
O1	0.5113 (4)	0.2902 (11)	0.7512 (13)	0.032 (2)	
C1	0.1856 (7)	0.6630 (14)	0.6881 (14)	0.026 (2)	
H1A	0.1894	0.7729	0.7479	0.032*	
C2	0.1231 (6)	0.6387 (14)	0.5429 (16)	0.027 (2)	
H2A	0.0876	0.7317	0.5050	0.032*	
C3	0.1165 (6)	0.4722 (15)	0.4594 (13)	0.023 (2)	
C4	0.1698 (7)	0.3367 (14)	0.5184 (14)	0.025 (2)	
H4A	0.1638	0.2236	0.4642	0.030*	
C5	0.2310 (7)	0.3651 (14)	0.6544 (16)	0.028 (2)	
H5A	0.2671	0.2725	0.6885	0.033*	
C6	0.2409 (6)	0.5321 (14)	0.7448 (17)	0.027 (2)	
C7	0.3094 (5)	0.5625 (12)	0.911 (3)	0.0244 (19)	
C8	0.4503 (7)	0.2840 (13)	0.8017 (14)	0.025 (2)	
C9	0.3368 (5)	0.7556 (12)	0.914 (3)	0.030 (2)	
H9A	0.3911	0.7610	0.9682	0.045*	
H9B	0.3081	0.8292	0.9779	0.045*	
H9C	0.3288	0.8004	0.7971	0.045*	

### Atomic displacement parameters (Å^2^)

**Table:**

	*U*^11^	*U*^22^	*U*^33^	*U*^12^	*U*^13^	*U*^23^
Br1	0.0269 (4)	0.0173 (4)	0.0201 (8)	−0.0037 (3)	0.0067 (17)	−0.0023 (5)
N3	0.039 (5)	0.013 (4)	0.057 (7)	0.000 (4)	0.026 (5)	−0.004 (5)
N1	0.019 (3)	0.015 (4)	0.083 (11)	0.001 (3)	0.011 (6)	−0.004 (6)
N2	0.033 (5)	0.009 (4)	0.043 (6)	0.001 (3)	0.018 (4)	−0.004 (4)
O1	0.029 (3)	0.037 (5)	0.038 (4)	−0.003 (3)	0.021 (4)	−0.001 (5)
C1	0.043 (6)	0.013 (4)	0.023 (5)	−0.003 (4)	0.008 (5)	−0.004 (4)
C2	0.023 (5)	0.019 (4)	0.039 (6)	−0.005 (4)	0.008 (5)	−0.003 (5)
C3	0.020 (4)	0.034 (6)	0.016 (4)	−0.001 (4)	0.008 (4)	0.005 (4)
C4	0.035 (5)	0.019 (5)	0.019 (5)	−0.008 (4)	0.002 (4)	0.004 (4)
C5	0.033 (5)	0.013 (4)	0.034 (6)	0.000 (4)	0.004 (5)	−0.005 (4)
C6	0.024 (5)	0.020 (5)	0.040 (6)	0.000 (4)	0.014 (5)	0.004 (4)
C7	0.021 (3)	0.016 (3)	0.036 (6)	0.003 (3)	0.005 (8)	−0.016 (6)
C8	0.046 (6)	0.007 (4)	0.024 (5)	0.007 (4)	0.009 (5)	−0.002 (4)
C9	0.022 (4)	0.011 (3)	0.060 (8)	−0.003 (3)	0.018 (8)	0.009 (8)

### Geometric parameters (Å, °)

**Table:**

Br1—C3	1.865 (10)	C2—C3	1.382 (16)
N3—C8	1.344 (14)	C2—H2A	0.9300
N3—H3A	0.8600	C3—C4	1.378 (15)
N3—H3B	0.8600	C4—C5	1.359 (15)
N1—C7	1.233 (11)	C4—H4A	0.9300
N1—N2	1.397 (9)	C5—C6	1.410 (15)
N2—C8	1.387 (12)	C5—H5A	0.9300
N2—H2B	0.8600	C6—C7	1.59 (2)
O1—C8	1.232 (13)	C7—C9	1.496 (12)
C1—C6	1.373 (15)	C9—H9A	0.9600
C1—C2	1.415 (16)	C9—H9B	0.9600
C1—H1A	0.9300	C9—H9C	0.9600
			
C8—N3—H3A	120.0	C4—C5—C6	121.4 (11)
C8—N3—H3B	120.0	C4—C5—H5A	119.3
H3A—N3—H3B	120.0	C6—C5—H5A	119.3
C7—N1—N2	115.2 (10)	C1—C6—C5	116.4 (11)
C8—N2—N1	118.1 (8)	C1—C6—C7	121.7 (9)
C8—N2—H2B	121.0	C5—C6—C7	121.8 (9)
N1—N2—H2B	121.0	N1—C7—C9	129.3 (10)
C6—C1—C2	123.1 (10)	N1—C7—C6	97.0 (12)
C6—C1—H1A	118.5	C9—C7—C6	109.2 (13)
C2—C1—H1A	118.5	O1—C8—N3	121.0 (9)
C3—C2—C1	117.8 (10)	O1—C8—N2	122.1 (9)
C3—C2—H2A	121.1	N3—C8—N2	116.9 (10)
C1—C2—H2A	121.1	C7—C9—H9A	109.5
C4—C3—C2	119.9 (10)	C7—C9—H9B	109.5
C4—C3—Br1	121.5 (8)	H9A—C9—H9B	109.5
C2—C3—Br1	118.6 (8)	C7—C9—H9C	109.5
C5—C4—C3	121.3 (10)	H9A—C9—H9C	109.5
C5—C4—H4A	119.4	H9B—C9—H9C	109.5
C3—C4—H4A	119.4		
			
C7—N1—N2—C8	−176.1 (14)	C4—C5—C6—C7	176.3 (13)
C6—C1—C2—C3	2.4 (17)	N2—N1—C7—C9	−20 (3)
C1—C2—C3—C4	0.1 (15)	N2—N1—C7—C6	101.6 (13)
C1—C2—C3—Br1	−179.9 (8)	C1—C6—C7—N1	−174.6 (12)
C2—C3—C4—C5	−2.4 (16)	C5—C6—C7—N1	9.4 (17)
Br1—C3—C4—C5	177.5 (9)	C1—C6—C7—C9	−38.6 (17)
C3—C4—C5—C6	2.4 (18)	C5—C6—C7—C9	145.3 (12)
C2—C1—C6—C5	−2.4 (17)	N1—N2—C8—O1	−175.3 (12)
C2—C1—C6—C7	−178.7 (12)	N1—N2—C8—N3	3.9 (16)
C4—C5—C6—C1	0.0 (17)		

### Hydrogen-bond geometry (Å, °)

**Table:**

*D*—H···*A*	*D*—H	H···*A*	*D*···*A*	*D*—H···*A*
N2—H2B···O1^i^	0.86	2.21	3.052 (12)	168
N3—H3A···O1^ii^	0.86	2.03	2.885 (13)	171
C9—H9A···O1^iii^	0.96	2.51	3.34 (2)	144

Symmetry codes: (i) −*x*+1, *y*+1/2, −*z*+3/2; (ii) −*x*+1, *y*−1/2, −*z*+3/2; (iii) −*x*+1, −*y*+1, −*z*+2.
